# Effectiveness of a Web-Based, Computer-Tailored, Pedometer-Based Physical Activity Intervention for Adults: A Cluster Randomized Controlled Trial

**DOI:** 10.2196/jmir.3402

**Published:** 2015-02-09

**Authors:** Sofie Compernolle, Corneel Vandelanotte, Greet Cardon, Ilse De Bourdeaudhuij, Katrien De Cocker

**Affiliations:** ^1^Physical Activity, Fitness and HealthDepartment of Movement and Sports SciencesGhent UniversityGentBelgium; ^2^Centre for Physical Activity StudiesInstitute for Health and Social Science ResearchCentral Queensland UniversityRockhamptonAustralia; ^3^Research Foundation FlandersB-1000 GhentBelgium

**Keywords:** physical activity, computer tailoring, Web-based intervention, cluster randomized controlled trial

## Abstract

**Background:**

Computer-tailored physical activity (PA) interventions delivered through the Internet represent a promising and appealing method to promote PA at a population level. However, personalized advice is mostly provided based on subjectively measured PA, which is not very accurate and might result in the delivery of advice that is not credible or effective. Therefore, an innovative computer-tailored PA advice was developed, based on objectively pedometer-measured PA.

**Objective:**

The study aim was to evaluate the effectiveness of a computer-tailored, pedometer-based PA intervention in working adults.

**Methods:**

Participants (≥18 years) were recruited between May and December 2012 from eight Flemish workplaces. These workplaces were allocated randomly to an intervention or control group. Intervention group participants (n=137) received (1) a booklet with information on how to increase their steps, (2) a non-blinded pedometer, and (3) an Internet link to request computer-tailored step advice. Control group participants (n=137) did not receive any of the intervention components. Self-reported and pedometer-based PA were assessed at baseline (T0), and 1 month (T1) and 3 months (T2) months post baseline. Repeated measures analyses of covariance were used to examine intervention effects for both the total sample and the at-risk sample (ie, adults not reaching 10,000 steps a day at baseline).

**Results:**

The recruitment process resulted in 274 respondents (response rate of 15.1%) who agreed to participate, of whom 190 (69.3%) belonged to the at-risk sample. Between T0 and T1 (1-month post baseline), significant intervention effects were found for participants’ daily step counts in both the total sample (*P*=.004) and the at-risk sample (*P*=.001). In the at-risk sample, the intervention effects showed a daily step count increase of 1056 steps in the intervention group, compared to a decrease of 258 steps in the control group. Comparison of participants’ self-reported PA revealed a significant intervention effect for time spent walking in the at-risk sample (*P*=.02). Intervention effects were still significant 3 months post baseline for participants’ daily step counts in both the total sample (*P*=.03) and the at-risk sample (*P*=.02); however, self-reported PA differences were no longer significant.

**Conclusions:**

A computer-tailored, pedometer-based PA intervention was effective in increasing both pedometer-based and self-reported PA levels, mainly in the at-risk participants. However, more effort should be devoted to recruit and retain participants in order to improve the public health impact of the intervention.

**Trial Registration:**

ClinicalTrials.gov: NCT02080585; https://clinicaltrials.gov/ct2/show/NCT02080585 (Archived by WebCite at http://www.webcitation.org/6VvQnRQSy).

## Introduction

Regular physical activity (PA) leads to multiple health benefits and reduces the risk of many chronic diseases [1-3]. Although these benefits are well established, most adults do not meet current PA recommendations [4,5]. International guidelines recommend at least 20 minutes of continuous, aerobic vigorous-intensity PA at least three times a week or at least 30 minutes of moderate-intensity PA five times a week [6]. An alternative guideline, proposed by Hatano [7] and frequently used in physical activity research, recommends at least 10,000 steps a day [8,9]. To stimulate adults in reaching these guidelines, different types of PA interventions have been developed in the past, such as pedometer-based interventions and computer-tailored interventions [10-12].

A recent meta-analysis, examining the effect of pedometer-based physical activity interventions, suggested that pedometer use has a moderate and positive effect on the increase of PA. Moreover, the effect was more pronounced when integrating 10,000 steps a day as the step goal [13]. Computer-tailored interventions have also been shown to be effective in supporting PA [14-18] and are offering several advantages. First, most computer-tailored interventions are Web-based interventions, which means that the advice can be requested online. Online interventions are shown to be appealing and feasible and have the ability to reach many people in a cost-effective manner at any time and location [19-21]. Second, computer-tailored interventions provide individualized advice, which is automatically generated based on participants’ answers to a predefined diagnostic questionnaire. Previous studies have shown that participants are more likely to increase their PA level when receiving tailored feedback, compared to generic feedback [12,22-24].

However, existing computer-tailored interventions also have limitations. Completing questionnaires is time-consuming, and self-reported PA data may have been influenced by response and recall biases [25]. Therefore, we developed Web-based, computer-tailored PA advice, based on participants’ objectively measured daily step counts [26]. Consequently, the assessment of baseline PA will be more accurate and participants will no longer need to complete an extensive questionnaire to assess their baseline PA level. This PA advice is relatively innovative, given that to date, only a few computer-tailored physical activity interventions were coupled with a personal activity monitor [14,27].

Feasibility of this Web-based, computer-tailored step advice was examined by De Cocker et al [26] in a pilot study. They conducted a randomized controlled trial among participants recruited through general practitioners (GPs) [26]. This demonstrated that the majority of the participants accepted the step advice well and that it was perceived as useful. While PA increased, no superior intervention effects on PA levels were found in the tailored condition, compared with the standard condition. This could be explained by three factors. First, the statistical power was limited, since the study sample at posttest was rather small (N=69). Only 20 participants provided objective pedometer data on both baseline and post-intervention measurements. Second, participants of the control condition also received a pedometer and step information during the study period; however, pedometers as a stand-alone intervention have shown to be effective in increasing step counts in adults as well [28]. Third, the pilot study assessed only pedometer-based and self-reported PA at two time points (baseline and 3 months post baseline), so it is not possible to examine the effect of the intervention immediately after requesting the advice.

To overcome these shortcomings, a new cluster randomized controlled trial was conducted to assess the effectiveness of Web-based tailored step advice in adults with (1) a larger sample, (2) a control group that did not receive any intervention component, and (3) three assessment points.

## Methods

### Participants and Study Design

This study used a cluster randomized controlled trial to evaluate the effects of a computer-tailored, pedometer-based PA intervention delivered through the Internet. Potential participants were recruited from “white-collar” workplaces, given that the majority of the employees in these workplaces were not physically active during the day. Managers of 18 workplaces were invited by email in three waves at different times of the year (to overcome seasonal effects). The first wave started in May 2012, the second wave in September 2012, and the third wave in December 2012. Eight workplaces, of which three schools (ie, secondary schools), three commercial organizations (ie, two software companies and one consulting company), and two non-profit organizations (ie, health insurance organizations), consented to participate. All employees of a single workplace were allocated at random to either the intervention or a waiting list control group by the first author using a computer-generated random list, in order to avoid contamination between employees receiving the intervention and those not receiving the intervention. Every wave contained at least one intervention and one control workplace, and both the intervention group and the control group contained at least one school, one commercial organization, and one non-profit organization. Subsequently, employees of the participating workplaces were recruited by email. Only Dutch-speaking employees between 18 and 65 years old and who had access to the Internet at work or at home were eligible. Interested employees could sign up by returning a confirmation email to the researchers. On receiving this information, a meeting was organized in each of the eight worksites to deliver all documents for baseline measurement (T0) to the participants, including an informed consent form, a blinded pedometer, an activity log, and a self-administered questionnaire. During this meeting, information was provided on how to use the pedometer, how to log PA activities, and how to answer the questionnaire. To reduce expectancy effects, researchers concealed information on the study’s focus, and asked participants to adhere to their usual PA pattern throughout the measurements. After 1 week, all measurement tools were collected, and average daily step counts were calculated. At this point, participants in the intervention condition received (1) a booklet with information on how to increase steps, (2) a non-blinded pedometer, which they could use for 3 months, and (3) a username, password, and the number of average daily steps, calculated by the researchers, so that participants could use this number when requesting the Web-based, computer-tailored step advice. Participants in the control condition did not receive any of the above mentioned intervention components. At 1 month and 3 months, all participants again received a blinded pedometer, which was worn for 1 week. When wearing the blinded-pedometer 1 month (T1) and 3 months (T2) post baseline, wearing the non-blinded pedometer was also allowed. Furthermore, the same self-reported questionnaire was used to measure PA level at T1 and T2. This study protocol was approved by the Ghent University Ethics Committee, and an informed consent was obtained from each participant before the study started.

See Multimedia Appendix 1 for the CONSORT-EHEALTH checklist [29].

### Computer-Tailored Intervention Website

The intervention website was developed based on previous computer-tailored interventions to increase PA in Flanders [16,30-32] and consists of two main parts, a Web-based questionnaire and computer-tailored step advice. The questionnaire assesses demographic variables, average daily steps, and psychosocial determinants towards 10,000 steps/day (Figure A in Multimedia Appendix 2; Table 1). The computer-tailored step advice includes feedback to help people reaching the PA recommendation of 10,000 steps/day. Three parts can be distinguished in the computer-tailored step advice. The first part consisted of a general introduction. The second part, included personalized feedback on the participants’ current number of steps. In this part, a schedule was provided on how they could reach the goal of 10,000 steps/day, based on participants’ preference of increasing their current step level with 500 or 1000 steps per week. The third part contained recommendations and suggestions to increase daily step counts (see Figures B-D in Multimedia Appendix 2). All three parts are based on the Theory of Planned Behavior [33] and the Transtheoretical Model [34]. The Theory of Planned Behavior is reflected by providing feedback on participants’ intentions, attitudes, self-efficacy, social support, knowledge, benefits, and barriers towards physical activity (see Table 1).

The Transtheoretical Model was used to adapt the content of the advice and the way of providing feedback to the stages of change. Precontemplators were mainly informed in an impersonal way about the idea of 10,000 steps, and its associated health benefits. Contemplators received the same information in a more personal way and were carefully informed that taking more steps might be beneficial for them. In the preparation stage, participants received less general information but were decisively asked to increase their daily steps. In the action stage, participants were encouraged in a supportive way to sustain their average daily steps. Some tips and tricks were provided to prevent relapse. In the maintenance stage, the feedback was limited to the message that they were doing well and should continue this way. If participants requested the computer-tailored step advice for a second time or more, progress feedback was provided by comparing their previous step level with their current step level. A more detailed description of the step advice can be found in De Cocker et al [26].

**Table 1:** Overview of the included psychosocial determinants.

Psychosocial determinant	Question	Answer possibilities	Example of the step advice
Intentions	Are you planning to step more within the upcoming 6 months?	Yes/no	You are planning to increase your daily step counts within 1 month. This is a good idea, as your current number of daily step counts is less than 10,000.
	Are you planning to step more within the upcoming month?		
Attitudes	I find it healthy to increase my daily step counts	Not agree/ sometimes agree, sometimes not agree/ Agree	You indicated that you did not find it healthy to increase your daily step counts. However, previous research has indicated that people who are physically active are less likely to develop cardiovascular diseases, obesity, hypertension, diabetes, osteoporosis, depression, cancer, etc
	I find it enjoyable to increase my daily step counts		
	I find it good to increase my daily step counts		
	I find it relaxing to increase my daily step counts		
Self-efficacy	Do you think you are able to increase your daily step counts on (1) most of the days in a usual week? (2) on days that you feel bad, tired, nervous, or depressed? (3) on days that you have a busy schedule?	I’m sure I can/ I think I can/ I’m sure I can’t	You are sure that you are not able to increase your daily step counts when you feel tired or depressed. However, it has been shown that being physically active reduces feelings of depression and exhaustion.
Social support	To what extent do you receive support from the following people to increase your daily step counts? Partner? Children (>12 years)? Friends?	Never/ Sometimes/ Often/ I do not have a partner, children, or friends	Studies have shown that people who have a partner to be physically active with, are more likely to sustain their physically active lifestyle. As you indicated that your partner is regularly physically active, it may be good idea to be physically active together.
	Are the following people regularly physically active? Partner? Children (>12 years)? Friends?	Yes/ No/ I do not have a partner, children, or friends	
Knowledge	Are you familiar with the use of a pedometer?	Yes/ No	You indicated that you are not familiar with the use of a pedometer. Therefore, you will find some general information about the use of a pedometer below.
Benefits	What is the most important benefit for you to increase your daily step counts?	To lose weight/ To feel less depressed/ To feel more attractive/ To get a better physical condition/ To meet new people/ To have fun/ To feel the kick of competition	Feeling less depressed as a consequence of an active lifestyle is important for you. This could be a good reason, as previous research has indicated that being physically active results in feeling less depressed.
Barriers	What are the two most important barriers for you to increase your daily step counts?	Lack of interest/ Lack of time/ Lack of self-discipline/ Lack of social support/ Lack of pleasure/ External factors, such as bad weather conditions, lack of money, lack of facilities/ Lack of a walking partner/ Lack of good health/ Being active makes me feel tired/ Having an injury	External factors hinder you from increasing your daily step counts. Nevertheless, being physically active does not have to be expensive, eg, walking, running and swimming are very cheap. Moreover, some sports do not require specific sport facilities.

### Measurements

#### Self-Reported Measurements

Demographic variables, PA, sitting time of the participants, and acceptability of the step advice were measured by means of a paper-based questionnaire. Demographic variables were assessed at baseline and included sex, age, height, weight, highest degree of education (primary or secondary education, college, university), health (very good, good, fair, bad, very bad), and place of residence (town, outskirts of town, village, or countryside). PA and sitting time were measured with the validated International Physical Activity Questionnaire (IPAQ), short version, at all time points [35]. In the questionnaire, both the frequency and duration of walking, moderate PA, vigorous PA, and time spent sitting during the past week were measured. Acceptability of step advice was examined by asking participants about the understandability, the logic, the practical use, and the length of the questionnaire. Furthermore, questions were asked about the relevance, the credibility, the understandability, the instructiveness, and the length of the step advice.

#### Objective Measurements

A blinded Omron HJ-203-ED pedometer, which showed good validity and reliability, and an activity log were used in the study [36]. The pedometer was equipped with a 7-day memory, allowing for daily steps to be automatically reset to zero at midnight. Participants were instructed to wear the pedometer around the neck, given that the least amount of error was observed for this wearing position [36]. Furthermore, the pedometer had to be worn for at least 5 days, including at least one weekend day, at all time points. Removal of the pedometer was permitted only during sleeping or water-based activities, such as bathing or swimming. The activity log was used to record the time and duration of non-walking activities (eg, swimming or cycling) and to document information about non-wearing of the pedometer (date and hours).

### Data Reduction

Participants’ baseline characteristics were described using means and standard deviations for quantitative variables and percentages for qualitative variables. Body mass index (BMI) was computed as self-reported weight in kilograms divided by self-reported square height in meters. Pedometer-based PA was expressed in steps/day and calculated for all participants with valid pedometer data (ie, if the total counts were >100, and the pedometer had been worn for at least 8 hours [37,38]) for at least 5 consecutive days [39]. Pedometer-data exceeding 20,000 steps/day were truncated as 20,000 to avoid unrealistically high data [40]. Self-reported total PA was computed by summing the time spent walking and doing moderate and vigorous PA in the last week. All self-reported physical activities were expressed in minutes/day. Data were cleaned as outlined in the IPAQ guidelines [41]. Both pedometer-based and self-reported PA data were log-transformed to correct for positive skewness (indicated by a significant Kolmogorov-Smirnov test) prior to further analyses.

### Statistical Analyses

Descriptive statistics were used to summarize participants’ baseline characteristics and to describe the acceptability of the step advice. Participants’ characteristics at baseline were compared by independent sample *t* tests for quantitative variables and by chi-square tests for qualitative variables to detect baseline differences between the control and the intervention group and to perform a drop-out analysis. Baseline characteristics that differed significantly between intervention and control group were used as covariates in further analyses. To determine what analyses should best be used to examine intervention effects, a three-level regression analysis was conducted (because of the hierarchical structure of the data) with assessment point at the first level, individual at the second level, and company at the third level. As the random part of the null model showed that the variance at the company level was not significantly different from zero (χ^2^_1_=3.06, *P=*.08), it is possible to examine intervention effects on PA behavior by conducting three 2x2 repeated measures analyses of covariance (ANCOVA) with time (two measurement moments) as within factor and condition (intervention group, control group) as between factor. Using these analyses also increases the interpretability of the outcomes. All repeated measures ANCOVAs were conducted separately for the total sample, as well as for the at-risk sample only (ie, adults not reaching 10,000 steps a day at baseline). Analyses were performed using MLwiN version 2.29 and IBM SPSS Statistics 21.0. The level of statistical significance was set at *P*≤.05; *P* values between .05 and .10 were considered borderline significant.

## Results

### Participant Characteristics, Response, and Attrition Rate

**Figure 1:**
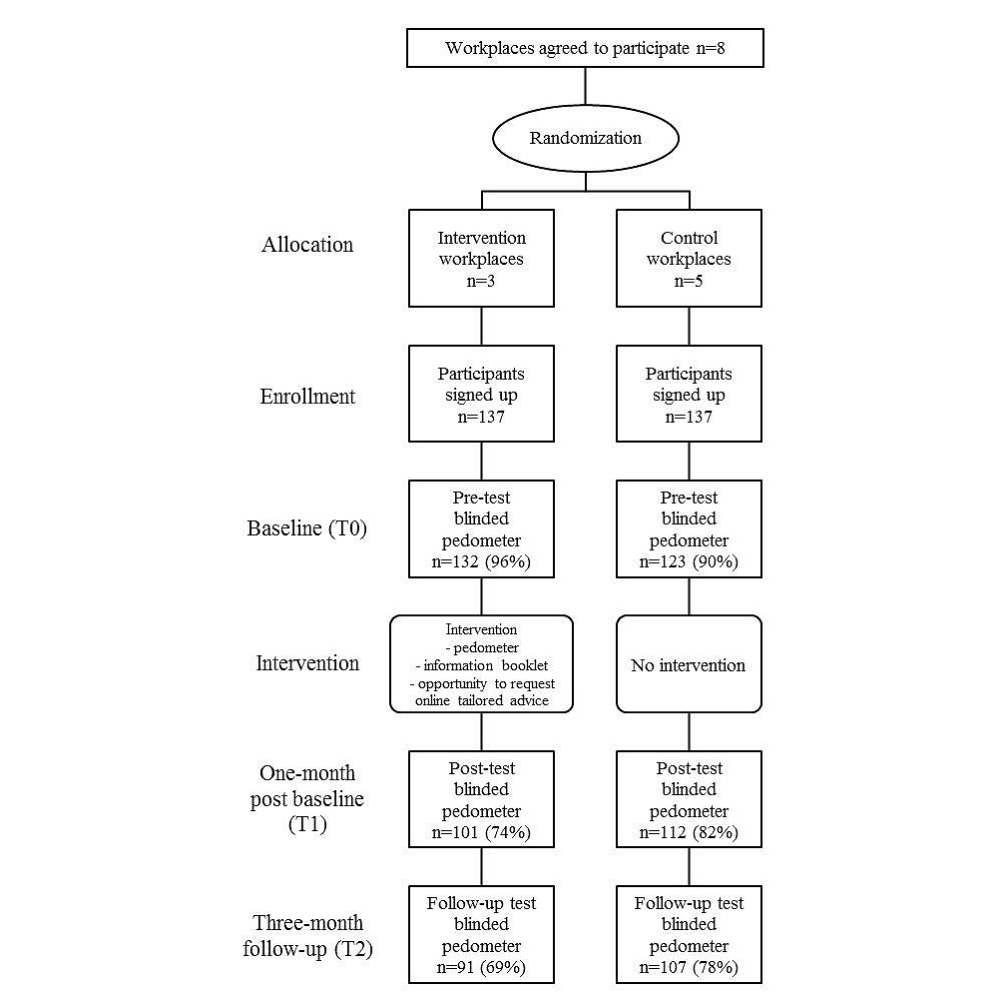
Participant flow through the study.

Figure 1 shows the flow of participants through the study. Invitation letters were sent to 1817 people, spread over eight workplaces. This recruitment process resulted in 274 respondents (response rate of 15%) who agreed to participate, of which 137 (50%) were allocated to the intervention group and 137 (50%) to the control group. Of the 137 intervention participants, 6 (4.4%) were in the precontemplation phase, 12 (8.8%) in the contemplation phase, 64 (47%) in the preparation stage, 35 (26%) in the action phase, and 20 (15%) did not provide information on their intentions. A total of 101 (74%) intervention participants and 112 (82%) control participants completed 1-month post baseline measurements, and 91 (66%) intervention participants and 107 (78%) control participants completed 3-month post baseline measurements. Finally, 91 intervention group participants and 107 control group participants had complete data. Drop-out analyses indicated that participants from the intervention group (χ^2^_1_=4.661, *P=*.03, two-tailed) and commercial companies (χ^2^_2_=27.087, *P*<.001, two-tailed) were more likely to drop out. No significant differences were found for demographic variables, pedometer-based PA, and self-reported PA between completers and dropouts (see Multimedia Appendix 3).

Baseline characteristics of the intervention and control group are presented in Table 2. The groups differed significantly at baseline in time spent sitting, with participants in the intervention group having a higher sitting time than participants in the control group (*P=*.01) (see Table 2). A trend of significance was observed for place of residence, with more participants living in a village or in the countryside in the intervention group (*P=*.06). No significant differences were found for the other demographic variables, pedometer-based and self-reported PA between intervention and control participants (see Multimedia Appendix 3).

**Table 2:** Comparison of baseline characteristics.

Characteristic			Intervention group	Control group	Group comparison	*P* value
**Demographic variable**						
	**Gender, n (%)**				χ^2^_1_=0.10	.76
		Male	50 (38.5)	45 (36.6)		
		Female	80 (61.5)	78 (63.4)		
	Age, mean (SD)		42.1 (11.4)	41.9 (10.7)	*t*_253_=0.20	.84
	BMI, mean (SD)		25.5 (4.9)	24.7 (3.8)	*t*_253_=1.37	.17
	**Education, n (%)**				χ^2^_2_=2.06	.36
		Primary/secondary	40 (31.2)	28 (23.1)		
		College	56 (43.8)	59 (48.8)		
		University	32 (25.0)	34 (28.1)		
	**Self-rated health, n (%)**				χ^2^_2_=0.09	.95
		Very good/good	101 (78.9)	96 (78.7)		
		Fair	22 (17.2)	22 (18.0)		
		Very bad/bad	5 (3.9)	4 (3.3)		
	**Place of residence, n (%)**				χ^2^_2_=5.79	.06^a^
		Town	25 (19.4)	30 (24.8)		
		Outskirts of town	47 (36.4)	56 (46.3)		
		Village/countryside	57 (44.2)	36 (29.8)		
	Pedometer-based PA (steps/day), mean (SD)		8329 (3869)	8324 (3926)	*t*_253_=0.14	.89
	**Self-reported PA and sedentary time (minutes/day)**					
		Sitting time	526.7 (163.7)	465.2 (186.1)	*t*_253_=2.82	.01^b^
		Walking	22.2 (65.2)	24.7 (77.9)	*t*_253_=0.04	.97
		Moderate PA	22.2 (26.2)	26.9 (39.6)	*t*_253_=0.82	.41
		Vigorous PA	9.9 (18.0)	9.0 (21.3)	*t*_253_=1.18	.24
		Total PA	53.6 (85.2)	55.7 (75.9)	*t*_253_=0.19	.85

^a^ *P*<.10.

^b^ *P*<.05.

### Acceptability of the Step Advice

The majority of the intervention group participants (118/137, 86%) did request the computer-tailored step advice. Most participants found the questions easily understandable (91/93, 98%) and that they progressed logically (88/90, 98%). Almost half of the participants considered the length of the questionnaire to be adequate (41/89, 46%), and 94% (84/89) of the participants had no problems answering the questions. The step advice itself was rated as interesting by 94% (82/87), as credible by 95% (84/88), as understandable by 96% (85/89) and as instructive by 80% (71/89). The only downside that was addressed was the length of the advice. More than half of the participants (80%, 71/89) found the advice too long.

### Changes in Physical Activity for the Total Sample

**Figure 2:**
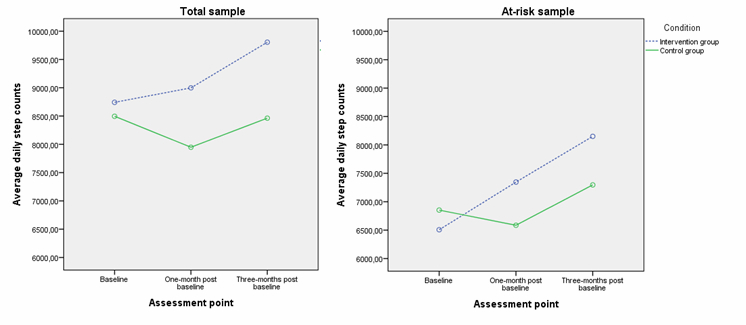
Change of average daily step counts of participants completing all three measurements from the total sample (N=168) and the at-risk sample (n=119).

Tables 3 to 5 present intervention effects for participants’ daily step counts, sitting time, walking time, moderate PA time, vigorous PA time, and total PA time. For the total group, comparison of participants’ pedometer-based PA revealed a significant intervention effect between T0 and T1 (*F*_1,192_=8.70, *P=*.004) and between T0 and T2 (*F*_1,176_=4.59, *P=*.03). Daily step counts in the intervention group increased from 8760 steps at T0 to 9235 at T1 (1 month later) and to 9484 at T2 (3 months later), while daily step counts of the control group decreased from 8628 at T0 to 8102 at T1, and to 8589 at T2. The percentage of individuals meeting the recommended guideline of 10,000 steps a day evolves from 36% (35/97) at T0 to 55% (36/65) at T1 and 65% (36/55) at T2 in the intervention group, and from 32% (30/93) at T0 to 35% (29/83) at T1 and 53% (37/70) at T2 in the control group. Figure 2 shows the change of average daily step counts of participants completing all three measurements. Comparison of participants’ self-reported PA indicated a trend of significance for moderate PA between T0 and T1 (*F*_1,161_=3.13, *P=*.08). Intervention group participants increased their moderate PA by 2.29 min/day, while control group participants decreased their moderate PA by 9.06 min/day. No significant intervention effects were found for time spent sitting, walking, being vigorously active, and for total PA.

### Changes in Physical Activity for the At-Risk Sample

For the at-risk sample, which included only the participants not reaching 10,000 steps at baseline (n=190, 69%), significant intervention effects on step counts were found between T0 and T1 (*F*_1,136_=11.98, *P=*.001) and between T0 and T2 (*F*_1,124_=5.54, *P=*.02). Daily step counts in the intervention group increased from 6697 steps at T0 to 7753 at T1 (1 month later) and to 8019 at T2 (3 months later), while daily step counts of the control group first decreased from 6898 at T0 to 6640 at T1 and subsequently increased to 7308 at T2. The percentage of individuals meeting the recommended guideline of 10,000 steps a day increased from 0% at T0 to 30% (16/53) at T1 and 34% (16/47) at T2 in the intervention group, and from 0% at T0 to 11% (8/76) at T1 and 28% (17/61) at T2 in the control group. No significant intervention effects were found between T1 and T2 (*F*_1,161_=.04, *P=*.84). Figure 2 represents the change of average daily step counts of at-risk participants completing all three measurements. Comparison of participants’ self-reported PA demonstrated a significant intervention effect for time spent walking between T0 and T1 (*F*_1,101_=3.06, *P*=.02). Both intervention and control group participants increased their walking time, though the increase in walking time was much higher in the intervention group (26.96 min/day) than in the control group (6.99 min/day). A trend for significance was found between T0 and T1 for moderate PA (*F*_1,107_=5.80, *P=*.08) and for total PA (*F*_1,96_=3.58, *P=*.06). From T0 to T1, intervention group participants increased their moderate PA by 4.68 min/day and their total PA by 33.93 min/day, while control group participants decreased their moderate PA by 9.89 min/day and their total PA by 3.48 min/day. Between T0 and T2, a trend was also found for vigorous PA (*F*_1,94_=3.05, *P=*.08). Vigorous PA increased by 5.47 min/day in the intervention group and decreased by 0.68 min/day in the control group. No intervention effects were found for time spent sitting.

**Table 3:** Effects on pedometer-based and self-reported PA in both conditions for the total sample and the at-risk sample (<10,000 steps at baseline) from T0 to T1^a^.

			Total sample					Risk sample				
				T0	T1	Time x Group			T0	T1	Time x Group	
			n	Mean (SD)	Mean (SD)	*F (df)*	*P*	n	Mean (SD)	Mean (SD)	*F (df)*	*P*
**Pedometer-based PA (steps/day)**												
		IG	96	8759.98 (3771.32)	9235.48 (4281.05)	8.698 (1,192)	.004^b^	65	6697.34 (1864.33)	7753.18 (3196.10)	11.977 (1,136)	.001^b^
		CG	99	8627.69 (3786.73)	8101.77 (3882.31)			74	6898.16 (1979.35)	6640.43 (2751.43)		
**Self-reported PA and sitting time (min/day)**												
	**Sitting time**											
		IG	83	512.11 (164.33)	511.20 (155.56)	0.003 (1,171)	.95	54	534.07 (163.11)	541.67 (142.90)	0.362 (1,116)	.55
		CG	91	460.91 (184.68)	464.73 (194.56)			65	497.46 (193.33)	498.46 (193.72)		
	**Walking**											
		IG	71	14.49 (22.86)	37.05 (92.52)	2.246 (1,153)	.14	45	12.49 (24.17)	39.48 (113.45)	5.801 (1,101)	.02^c^
		CG	85	26.17 (51.93)	42.37 (86.66)			59	12.56 (16.60)	19.55 (22.35)		
	**Moderate PA**											
		IG	81	23.30 (28.11)	25.59 (36.85)	3.133 (1,161)	.08^d^	52	16.94 (24.05)	21.62 (34.70)	3.057 (1,107)	.08^d^
		CG	83	24.94 (36.21)	15.43 (20.08)			58	19.45 (37.70)	9.56 (11.27)		
	**Vigorous PA**											
		IG	84	10.64 (17.80)	9.13 (15.20)	0.422 (1,169)	.52	54	6.88 (13.57)	6.67 (12.70)	0.534 (1,114)	.47
		CG	88	9.76 (23.10)	6.78 (13.48)			63	5.87 (18.68)	3.68 (8.13)		
	**Total PA**											
		IG	70	49.00 (52.11)	73.68 (106.35)	1.989 (1,145)	.16	44	36.87 (52.74)	70.80 (124.19)	3.575 (1,96)	.06^d^
		CG	78	56.11 (72.67)	55.47 (64.56)			55	35.72 (49.60)	32.24 (27.81)		

^a^IC=intervention group, CG=control group.

^b^ *P*<.01.

^c^ *P*<.05.

^d^.05<*P*<.10.

**Table 4:** Effects on pedometer-based and self-reported PA in both conditions for the total sample and the at-risk sample (<10,000 steps at baseline) from T1 to T2^a^.

			Total sample					Risk sample				
				T1	T2	Time x Group			T1	T2	Time x Group	
			n	Mean (SD)	Mean (SD)	*F (df)*	*P*	n	Mean (SD)	Mean (SD)	*F (df)*	*P*
**Pedometer-based PA (steps/day)**												
		IG	78	8823.67 (3956.87)	9629.90 (4971.40)	0.003 (1,167)	.96	52	7298.08 (2654.68)	8092.41 (4068.30)	0.041 (1,116)	.84
		CG	92	8184.75 (3972.33)	8679.54 (4420.83)			67	6622.99 (2857.11)	7342.06 (3822.00)		
**Self-reported PA and sitting time (min/day)**												
	**Sitting time**											
		IG	60	514.31 (156.97)	463.10 (156.01)	0.062 (1,140)	.80	37	554.44 (131.22)	488.47 (135.07)	0.299 (1,91)	.59
		CG	83	461.23 (196.16)	413.95 (197.17)			57	499.45 (196.35)	462.09 (202.54)		
	**Walking**											
		IG	51	35.42 (50.86)	37.87 (55.20)	0.039 (1,127)	.84	33	29.07 (52.89)	30.97 (56.82)	<0.001 (1,82)	1.00
		CG	79	43.42 (89.54)	47.74 (71.75)			52	18.88 (21.59)	39.52 (50.35)		
	**Moderate PA**											
		IG	56	27.28 (32.17)	32.61 (38.16)	1.089 (1,131)	.30	35	22.33 (23.71)	32.24 (38.52)	1.709 (1,85)	.20
		CG	78	16.62 (20.24)	37.67 (60.26)			53	10.75 (11.33)	32.83 (55.47)		
	**Vigorous PA**											
		IG	54	7.94 (12.50)	12.29 (19.36)	0.779 (1,130)	.38	34	5.55 (10.20)	10.21 (18.43)	1.433 (1,84)	.23
		CG	79	8.83 (17.68)	10.84 (18.82)			53	4.03 (8.66)	6.02 (14.28)		
	**Total PA**											
		IG	43	68.84 (81.74)	82.48 (80.06)	0.714 (1,114)	.40	28	55.51 (80.35)	79.54 (86.90)	0.943 (1,74)	.34
		CG	74	63.08 (80.74)	92.52 (100.54)			49	33.71 (28.30)	80.55 (96.10)		

^a^IC=intervention group, CG=control group.

**Table 5:** Effects on pedometer-based and self-reported PA in both conditions for the total sample and the at-risk sample (<10,000 steps at baseline) from T0 to T2^a^.

			Total sample					Risk sample				
				T0	T2	Time x Group			T0	T2	Time x Group	
			n	Mean (SD)	Mean (SD)	*F*	*P*	n	Mean (SD)	Mean (SD)	*F*	*P*
**Pedometer-based PA (steps/day)**												
		IG	86	8418.95 (3843.53)	9483.86 (4875.34)	4.587 (1,176)	.03^b^	59	6443.42 (1917.63)	8019.24 (3997.34)	5.536 (1,124)	.02^b^
		CG	93	8613.87 (3774.78)	8589.15 (4379.61)			68	6805.71 (2074.47)	7308.22 (3803.62)		
**Self-reported PA and sitting time (min/day)**												
	**Sitting time**											
		IG	69	525.58 (153.76)	467.76 (168.87)	0.010 (1,153)	.92	45	559.56 (134.90)	501.78 (152.09)	0.005 (1,101)	.95
		CG	87	463.02 (185.45)	411.26 (197.00)			59	507.71 (183.27)	460.25 (201.61)		
	**Walking**											
		IG	51	28.05 (88.35)	35.16 (52.49)	1.091 (1,127)	.30	38	12.50 (23.27)	31.45 (56.88)	0.847 (1,86)	.36
		CG	79	27.37 (54.01)	47.37 (72.60)			51	12.24 (15.65)	37.87 (50.84)		
	**Moderate PA**											
		IG	62	25.38 (26.76)	32.37 (37.68)	1.233 (1,138)	.27	40	18.50 (23.94)	30.38 (36.23)	1.850 (1,90)	.17
		CG	79	30.88 (42.50)	38.44 (60.04)			53	26.13 (44.55)	33.95 (55.84)		
	**Vigorous PA**											
		IG	63	10.00 (17.20)	13.73 (21.58)	1.893 (1,141)	.17	41	5.51 (11.31)	10.98 (20.89)	3.053 (1,94)	.08^c^
		CG	81	11.31 (24.30)	10.69 (18.44)			56	6.71 (19.73)	6.03 (14.02)		
	**Total PA**											
		IG	52	62.72 (113.96)	81.62 (78.03)	0.139 (1,120)	.71	35	34.18 (47.64)	76.02 (80.73)	0.329 (1,80)	.17
		CG	71	60.55 (74.29)	90.59 (100.21)			48	39.32 (51.21)	79.63 (95.13)		

^a^IC=intervention group, CG=control group.

^b^ *P*<.05.

^c^.05<*P*<.10.

## Discussion

### Principal Findings

The aim of this study was to evaluate the effectiveness of a Web-based, tailored, pedometer-based PA intervention in adults. The results revealed that the combination of the pedometer, the information booklet, and the computer-tailored step advice has the potential to enhance objectively measured daily step counts in both the total sample and the at-risk sample. Although, the intervention effects were noticeable in both samples, differences were much more pronounced in the at-risk sample. Effects on subjectively measured physical activity were rather limited, with only one significant intervention effect found for self-reported time spent walking in the at-risk sample. This highlights the need for objective measurement.

The findings of this study add new evidence for the effectiveness of computer-tailored PA interventions. Previous reviews [12,42] showed that computer-tailored PA interventions demonstrated mixed effects. Whereas some studies reported significant increases in PA [14-18], others did not yield significant improvements [43-45]. However, it should be noted that all these interventions formulated feedback based on self-reported PA data. Self-reported PA is prone to reporting biases, most often in the direction of overestimating physical activity [46]. Consequently, people might receive feedback indicating that they are doing enough PA, whereas in reality, they are not meeting the PA guidelines. Therefore, integrating objectively measured PA in a computer-tailored intervention is of added value, as it will result in more accurate feedback with a higher personal relevance. As such, the advice will have a higher credibility and consequently be more effective in changing behavior. To our knowledge, only one other study also used objective PA measures [27]. In this study, participants received a personal activity monitor (PAM) combined with tailored PA advice. However, no significant improvements in PA levels were found, which is in contrast with our results. A possible explanation could be that because the attractiveness of the activity advice in that study was rather low (only 39% of the users found the advice appealing), it was not encouraging enough for participants to become more active; whereas, the acceptability of the step advice was rated more positively in our study, with more than 90% of the participants rated the advice as interesting, understandable, and credible.

### Strengths and Limitations

In the pilot study of De Cocker et al [26], participants were recruited through general practitioners (GP). This was considered as a favorable dissemination channel, since GPs have personal face-to-face contact with their patients, and GPs are a credible health information source [47]. Unfortunately, this recruitment strategy was not as successful as expected, since only 6.2% of those approached consented to participate. Therefore, we used another recruitment strategy, in which employers and employees of a convenience sample of white-collar workplaces were invited. This recruitment strategy appeared to be more effective, given that more than twice as many people (15%) agreed to participate. This could possibly be explained by the fact that employers and employees experienced more social support than people invited by their GP, since all employers and employees within a company were invited to participate. Nonetheless, although the response rate was higher than in the study by De Cocker et al [26], it should be noted that still relatively few people enrolled for the intervention, in comparison with previous computer-tailored intervention studies [20]. Moreover, the recruitment through white-collar workplaces resulted in a selection bias with more highly educated people being involved in the study, which is in line with the outcomes of previous reviews that indicated that mainly higher educated people participate in online interventions [20,48]. This hampers the generalizability of the study results for those who are not as well educated.

An unexpectedly high attrition rate was observed in the intervention group as well as in the control group. Almost half of the intervention group participants (43%) and over one third (34%) of the control group participants dropped out at T2, which is relatively high in comparison with the attrition rates reported in recent reviews. In the review of Joseph et al assessing Internet-based PA interventions, an average attrition rate of 22% was reported [10]. In the meta-analysis of Davies et al [49], in which the overall effect size of PA interventions delivered through the Internet was calculated, an average attrition rate of 20% was found. Nevertheless, when considering only intervention groups, the average attrition rate reported by Davies et al was higher, more specifically 23% [49]. This higher percentage of dropouts in the intervention group is in line with our results and may be due to the fact that many intervention websites are not designed for people to be visited more than once. The main reason to revisit the step advice website is to see how one’s PA level has been changed, but it is unlikely that participants will do this without specific prompts to return to the website.

Additionally, beyond the computer-tailored module, the website did not have many interactive features, although many studies indicated that a high level of interactivity is needed to keep people interested and engaged with online interventions [31,50]. Due to the higher than expected attrition rate, the absolute sample size at 3 months post baseline is rather low, especially concerning the self-reported PA data. This results in a restricted statistical power, which could probably explain the lack of intervention effects at 3 months post baseline on the self-reported PA data. Moreover, it should be noted that the intervention group received different components (ie, pedometer, information booklet, and computer-tailored step advice). However, our study design does not allow us to determine whether all components are effective and whether their combination is necessary. Future studies should separate the different intervention components, in order to assess their individual impact. Finally, the relatively short study duration must be taken into account when interpreting the results. It may be that the intervention effects will disappear over time. As stated in the socio-ecological model, PA is the result of a complex interaction between individual level factors, and sociocultural, political, physical, and economic environmental factors [51]. Therefore, it is plausible that an individual initially changes their behavior as a result of an intervention but then relapses to previous unhealthy behavior as a consequence of the unchanged, obesogenic environment. Hence, multilevel interventions, where individual components are supported by environmental intervention components, have gained importance and should be evaluated.

### Conclusions

In conclusion, this is the first study to examine the effectiveness of integrating Web-based, computer-tailored, pedometer-based step advice in a physical activity intervention. The use of objective measures in providing tailored advice seems promising, given that this Web-based, computer-tailored, pedometer-based PA intervention showed significant effects on both pedometer-based PA and self-reported PA. However, more efforts should be devoted to recruit and retain participants in order to improve the public health impact of the intervention. Furthermore, we would like to encourage future research to include the assessment of other objective health risk factors (eg, blood pressure, BMI) in order to evaluate the intervention’s impact on health.

## Multimedia Appendix 1

CONSORT-EHEALTH checklist V1.6.2 [51].

## Multimedia Appendix 2

Screenshots of the Web-based, computer-tailored, pedometer-based physical activity advice.

## Multimedia Appendix 3

Comparison of completers and noncompleters.

## Abbreviations

ANCOVA: analysis of covariance
BMI: Body Mass Index
GP: general practitioner
IPAQ: International Physical Activity Questionnaire
PA: physical activity
