# Supplementary material for: Effectiveness of a Web-Based, Computer-Tailored, Pedometer-Based Physical Activity Intervention for Adults: A Cluster Randomized Controlled Trial
Source: J Med Internet Res. 2015 Feb 9;17(2):e38. doi: 10.2196/jmir.3402 (PMC4342625; doi:10.2196/jmir.3402)
Supplement: Supplementary file 1 [file jmir_v17i2e38_app1.pdf]

CONSORT-EHEALTH Checklist V1.6.2 Report

(based on CONSORT-EHEALTH V1.6), available at [http://tinyurl.com/consort-ehealth-v1-6].

3402

Date completed

3/16/2014 3:54:12

by

Sofie Compernolle

Effectiveness of a web-based, computer-tailored, pedometer-based physical activity intervention for adults: a cluster-randomized controlled trial

TITLE

1a-i) Identify the mode of delivery in the title

Yes, see title: Effectiveness of a web-based, computer-tailored, pedometer-based physical activity intervention for adults: a cluster-randomized controlled trial

1a-ii) Non-web-based components or important co-interventions in title

Yes, intervention participants also received a pedometer. This is reflected in the title: 'pedometer-based'.

1a-iii) Primary condition or target group in the title

Yes, the title states that 'adults' are the target group.

ABSTRACT

1b-i) Key features/functionalities/components of the intervention and comparator in the METHODS section of the ABSTRACT

Intervention group participants (n=137) received (1) a booklet with information on how to increase their steps, (2) a non-blinded pedometer and (3) an Internet link to request computer-tailored step advice. Control group participants (n=137) did not receive any of the above mentioned intervention components.

1b-ii) Level of human involvement in the METHODS section of the ABSTRACT

an Internet link to request computer-tailored step advice'

1b-iii) Open vs. closed, web-based (self-assessment) vs. face-to-face assessments in the METHODS section of the ABSTRACT

Participants (≥18 years) were recruited between May 2012 and December 2012 from eight Flemish workplaces. '
'Self-reported (IPAQ) and pedometer-based PA were assessed'

1b-iv) RESULTS section in abstract must contain use data

The recruitment process resulted in 274 respondents (response rate of 15.1%) who agreed to participate.'

1b-v) CONCLUSIONS/DISCUSSION in abstract for negative trials

Primary outcome: A computer-tailored, pedometer-based PA intervention was effective in increasing both pedometer-based, and self-reported PA levels.
Negative: However, more efforts should be devoted to recruit and retain participants in order to improve the public health impact of the intervention.

INTRODUCTION

2a-i) Problem and the type of system/solution

Page 4: 'existing computer-tailored interventions also have limitations. Completing questionnaires is time-consuming and, self-reported PA data may have been influenced by response and recall biases [17]. Therefore, we developed a web-based computer-tailored PA advice, based on participants' objectively measured daily step counts [18]. Consequently, the assessment of baseline PA will be more accurate and participants will no longer need to complete an extensive questionnaire to assess their baseline PA level.'

2a-ii) Scientific background, rationale: What is known about the (type of) system

Page 4-5: Feasibility of this web-based computer-tailored step advice was examined by De Cocker et al. (2012) in a pilot study. In this pilot study, a randomized controlled trial was conducted among participants recruited through general practitioners (GPs) [18]. This demonstrated that the majority of the participants accepted the step advice well, and that is was perceived as useful. While PA increased, no superior intervention effects on PA levels were found in the tailored condition, compared with the standard condition. This could be explained by three factors. Firstly, the statistical power was limited, since the study sample at posttest was rather small (n=69). Only 20 participants provided objective pedometer data on both baseline and post intervention measurements. Secondly, participants of the control condition also received a pedometer and step information during the study period; however pedometers as stand-alone intervention have shown to be effective in increasing step counts in adults as well [19]. Thirdly, the pilot study only assessed pedometer-based and self-reported PA at two time points (baseline and three-months post baseline), whereby it is not possible to examine the effect of the intervention immediately after requesting the advice.
To overcome these shortcomings,... '

METHODS

3a) CONSORT: Description of trial design (such as parallel, factorial) including allocation ratio

Page 5: To overcome these shortcomings, a new cluster-randomized controlled trial was conducted to assess the effectiveness of the online-tailored step advice in adults with 1) a larger sample, 2) a control group that did not receive any intervention component and 3) three assessment points.

3b) CONSORT: Important changes to methods after trial commencement (such as eligibility criteria), with reasons

Not relevant, methods were not changed.

3b-i) Bug fixes, Downtimes, Content Changes

Not relevant, no important changes were made.

4a) CONSORT: Eligibility criteria for participants

Page 5: 'Only Dutch speaking employees between 18 and 65 years old, who had access to the Internet at work or at home, were eligible.'

4a-i) Computer / Internet literacy

Page 5: 'Only Dutch speaking employees between 18 and 65 years old, who had access to the Internet at work or at home, were eligible. Interested employees could sign up by returning a confirmation e-mail to the researchers.'

4a-ii) Open vs. closed, web-based vs. face-to-face assessments:

Page 5: 'An invitation e-mail with study information was sent to managers of 18 white-collar workplaces in three waves at different times of the year to overcome seasonal effects. The first wave started in May 2012, the second wave in September 2012 and the third wave in December 2012. Eight workplaces, of which three schools, three commercial organizations, and two non-profit organizations, consented to participate. All employees of a single workplace were allocated at random to either the intervention or a waiting list control group, in order to avoid contamination between employees receiving the intervention and those who were not receiving the intervention. Every wave contained at least one intervention and one control workplace. Subsequently, employees of the participating workplaces were recruited by e-mail. '

4a-iii) Information giving during recruitment

Page 5: 'employees of the participating workplaces were recruited by e-mail. '
Page 5-6: 'On receiving this information, a meeting was organized in each of the eight worksites to deliver all documents for baseline measurement (T0) to the participants, including an informed consent form, a blinded pedometer, an activity log and a self-administered questionnaire. During this meeting, information was provided on how to use the pedometer, how to log PA activities and how to answer the questionnaire. Moreover, the participants were asked to adhere to their usual PA pattern throughout the baseline measurement.'

4b) CONSORT: Settings and locations where the data were collected

Page 7: 'Demographic variables, PA and sitting time of the participants were measuredby means of a paper-based questionnaire. '
Page 7-8: 'A blinded Omron HJ-203-ED pedometer, which showed good validity and reliability, and an activity log were used in the study [27]. The pedometer was equipped with a seven-day memory, allowing for daily steps to be automatically reset to zero at midnight. Participants were instructed to wear the pedometer around the neck, given that the least amount of error was observed for this wearing position [27]. Furthermore, the pedometer had to be worn for at least five days, including at least one weekend day, at all time points. Removal of the pedometer was only permitted during sleeping or water-based activities, such as bathing or swimming. The activity log was used to record the time and duration of non-walking activities (e.g., swimming or cycling) and to document information about non-wearing of the pedometer (date and hours). '

4b-i) Report if outcomes were (self-)assessed through online questionnaires

Page 7: 'Demographic variables, PA and sitting time of the participants were measuredby means of a paper-based questionnaire'

4b-ii) Report how institutional affiliations are displayed

All correspondence and the website had the logo of the Ghent University.

5) CONSORT: Describe the interventions for each group with sufficient details to allow replication, including how and when they were actually administered

5-i) Mention names, credential, affiliations of the developers, sponsors, and owners

Page 6: 'The intervention website was developed based on previous computer-tailored interventions to increase PA in Flanders [20-23], '
Page 4: 'Therefore, we developed a web-based computer-tailored PA advice, based on participants' objectively measured daily step counts [18]'

5-ii) Describe the history/development process

We did not explain the history in detail, as this was done in a previous paper: 'A more detailed description of the step advice can be found in De Cocker et al. (2012) [18].'
A general explanation was given on page 6-7: 'The intervention website was developed based on previous computer-tailored interventions to increase PA in Flanders [20-23], and consists of two main parts, an online questionnaire and a computer-tailored step advice. The online questionnaire assesses demographic variables, average daily steps and psychosocial determinants towards 10,000 steps/day (see Multimedia Appendix 1, Figure A). The computer-tailored step advice includes feedback to help people reaching the PA recommendation of 10,000 steps/day. Three parts can be distinguished in the computer-tailored step advice: a general introduction, personalized feedback on the participants' current number of steps, and recommendations and suggestions to increase daily step counts (see Multimedia Appendix 1, Figure B-D). All three parts are based on the Theory of Planned Behavior [24] and the Transtheoretical Model [25].'

5-iii) Revisions and updating

Page 6: 'The intervention website was developed based on previous computer-tailored interventions to increase PA in Flanders [20-23], '

5-iv) Quality assurance methods

Page 7: 'A blinded Omron HJ-203-ED pedometer, which showed good validity and reliability, and an activity log were used in the study [27].'

5-v) Ensure replicability by publishing the source code, and/or providing screenshots/screen-capture video, and/or providing flowcharts of the algorithms used

Screenshots were added as a Multimedia Appendix.

5-vi) Digital preservation

The URL was not provided, as the step advice was only available during the study period. Given the positive outcomes of this study, the step advice will be made publicly available in the upcoming months, but the URL is not known yet.

5-vii) Access

Page 6: 'At this point, participants in the intervention condition received (1) a booklet with information on how to increase steps, (2) a non-blinded pedometer, which they could use for three months, and (3) a username, a password and the amount of average daily steps, calculated by the researchers, so that participants could use this number when requesting the online computer-tailored step advice. '

5-viii) Mode of delivery, features/functionalities/components of the intervention and comparator, and the theoretical framework

Page 6: 'participants in the intervention condition received (1) a booklet with information on how to increase steps, (2) a non-blinded pedometer, which they could use for three months, and (3) a username, a password and the amount of average daily steps, calculated by the researchers, so that participants could use this number when requesting the online computer-tailored step advice. '
Page 6: 'The online questionnaire assesses demographic variables, average daily steps and psychosocial determinants towards 10,000 steps/day (see Multimedia Appendix 1, Figure A). The computer-tailored step advice includes feedback to help people reaching the PA recommendation of 10,000 steps/day. '
Page 6: 'All three parts are based on the Theory of Planned Behavior [24] and the Transtheoretical Model [25]. '

5-ix) Describe use parameters

No information was given to the participants regarding doses, timing, frequency, because we want to give participants freedom of choice.

5-x) Clarify the level of human involvement

Page 6: 'computer-tailored interventions'. As the intervention was computer-tailored, there was no human involvement.

5-xi) Report any prompts/reminders used

We did not use prompts or reminders, as we wanted to give the participants freedom of choice.

5-xii) Describe any co-interventions (incl. training/support)

Page 6: 'participants in the intervention condition received (1) a booklet with information on how to increase steps, (2) a non-blinded pedometer, which they could use for three months, and (3) a username, a password and the amount of average daily steps, calculated by the researchers, so that participants could use this number when requesting the online computer-tailored step advice. '

6a) CONSORT: Completely defined pre-specified primary and secondary outcome measures, including how and when they were assessed

Page 6: 'On receiving this information, a meeting was organized in each of the eight worksites to deliver all documents for baseline measurement (T0) to the participants, including an informed consent form, a blinded pedometer, an activity log and a self-administered questionnaire. During this meeting, information was provided on how to use the pedometer, how to log PA activities and how to answer the questionnaire. Moreover, the participants were asked to adhere to their usual PA pattern throughout the baseline measurement. After one week, all measurement tools were collected, and average daily step counts were calculated. At this point, participants in the intervention condition received (1) a booklet with information on how to increase steps, (2) a non-blinded pedometer, which they could use for three months, and (3) a username, a password and the amount of average daily steps, calculated by the researchers, so that participants could use this number when requesting the online computer-tailored step advice. Participants in the control condition did not receive any of the above mentioned intervention components. One and three months later, all participants again received a blinded pedometer, which was worn for one week. '
Page 7: 'Self-reported measurements
Demographic variables, PA and sitting time of the participants were measuredby means of a paper-based questionnaire. Demographic variables were assessed at baseline and included sex, age, height, weight, highest degree of education (primary or secondary education, college, university), health (very good, good, fair, bad, very bad) and place of residence (town, outskirts of town, village or countryside). PA and sitting time were measured with the validated International Physical Activity Questionnaire (IPAQ), short version, at all time points [26]. In the questionnaire, both the frequency and duration of walking, moderate PA, vigorous PA and time spent sitting during the past week were measured.
Objective measurements
A blinded Omron HJ-203-ED pedometer, which showed good validity and reliability, and an activity log were used in the study [27]. The pedometer was equipped with a seven-day memory, allowing for daily steps to be automatically reset to zero at midnight. Participants were instructed to wear the pedometer around the neck, given that the least amount of error was observed for this wearing position [27]. Furthermore, the pedometer had to be worn for at least five days, including at least one weekend day, at all time points. Removal of the pedometer was only permitted during sleeping or water-based activities, such as bathing or swimming. The activity log was used to record the time and duration of non-walking activities (e.g., swimming or cycling) and to document information about non-wearing of the pedometer (date and hours). '

6a-i) Online questionnaires: describe if they were validated for online use and apply CHERRIES items to describe how the questionnaires were designed/deployed

Not applicable, as we used paper-based questionnaires in our study.

6a-ii) Describe whether and how "use" (including intensity of use/dosage) was defined/measured/monitored

No information was provided on 'use', as the intervention website was not really designed to be visited more than once.

6a-iii) Describe whether, how, and when qualitative feedback from participants was obtained

Qualitative information regarding the step advice was provided in a previous study.

6b) CONSORT: Any changes to trial outcomes after the trial commenced, with reasons

Not applicable for this study.

7a) CONSORT: How sample size was determined

7a-i) Describe whether and how expected attrition was taken into account when calculating the sample size

No sample size calculations were conducted a priori.

7b) CONSORT: When applicable, explanation of any interim analyses and stopping guidelines

Not applicable.

8a) CONSORT: Method used to generate the random allocation sequence

Page 5: 'All employees of a single workplace were allocated at random to either the intervention or a waiting list control group, in order to avoid contamination between employees receiving the intervention and those who were not receiving the intervention. '

8b) CONSORT: Type of randomisation; details of any restriction (such as blocking and block size)

Page 5: 'An invitation e-mail with study information was sent to managers of 18 white-collar workplaces in three waves at different times of the year to overcome seasonal effects. The first wave started in May 2012, the second wave in September 2012 and the third wave in December 2012. Eight workplaces, of which three schools, three commercial organizations, and two non-profit organizations, consented to participate. All employees of a single workplace were allocated at random to either the intervention or a waiting list control group, in order to avoid contamination between employees receiving the intervention and those who were not receiving the intervention. Every wave contained at least one intervention and one control workplace.
'

9) CONSORT: Mechanism used to implement the random allocation sequence (such as sequentially numbered containers), describing any steps taken to conceal the sequence until interventions were assigned

Page 5: 'An invitation e-mail with study information was sent to managers of 18 white-collar workplaces in three waves at different times of the year to overcome seasonal effects. The first wave started in May 2012, the second wave in September 2012 and the third wave in December 2012. Eight workplaces, of which three schools, three commercial organizations, and two non-profit organizations, consented to participate. All employees of a single workplace were allocated at random to either the intervention or a waiting list control group, in order to avoid contamination between employees receiving the intervention and those who were not receiving the intervention. Every wave contained at least one intervention and one control workplace.
'

10) CONSORT: Who generated the random allocation sequence, who enrolled participants, and who assigned participants to interventions

Page 5: 'An invitation e-mail with study information was sent to managers of 18 white-collar workplaces in three waves at different times of the year to overcome seasonal effects. The first wave started in May 2012, the second wave in September 2012 and the third wave in December 2012. Eight workplaces, of which three schools, three commercial organizations, and two non-profit organizations, consented to participate. All employees of a single workplace were allocated at random to either the intervention or a waiting list control group, in order to avoid contamination between employees receiving the intervention and those who were not receiving the intervention.'
Page 5: 'Interested employees could sign up by returning a confirmation e-mail to the researchers'

11a) CONSORT: Blinding - If done, who was blinded after assignment to interventions (for example, participants, care providers, those assessing outcomes) and how

11a-i) Specify who was blinded, and who wasn't

It was not possible to blind the participants, as intervention participants received the intervention after one week, and control participants received the intervention after three months.

11a-ii) Discuss e.g., whether participants knew which intervention was the "intervention of interest" and which one was the "comparator"

Participants were not aware if they received the intervention of interest or the comparator.

11b) CONSORT: If relevant, description of the similarity of interventions

Not relevant

12a) CONSORT: Statistical methods used to compare groups for primary and secondary outcomes

Page 8-9: 'Participants' characteristics at baseline were compared by independent sample t-tests for quantitative variables and by chi-square tests for qualitative variables to detect baseline differences between the control and the intervention group, and to do a drop-out analysis. Baseline characteristics that differed significantly between intervention and control group were used as covariates in further analyses. To determine what analyses should best be used to examine intervention effects, a three-level regression analysis was conducted (because of the hierarchical structure of the data) with assessment point at the first level, individual at the second level and company at the third level. As the random part of the null model showed that the variance at the company level was not significantly different from zero ($\chi^2 = 3.06$, df = 1, P = .08) it is possible to examine intervention effects on PA behavior by conducting three 2x2 repeated measures analyses of covariance (ANCOVA) with time (two measurement moments) as within factor and condition (intervention group, control group) as between factor. Using these analyses also increases the interpretability of the outcomes. All repeated measures ANCOVA's were conducted separately for the total sample, as well as for the at risk sample only (i.e. adults not reaching 10,000 steps a day at baseline). Analyses were performed using MLwiN version 2.29 and IBM SPSS Statistics 21.0. The level of statistical significance was set at $P \leq 0.05$. P-values between 0.05 and 0.10 were considered borderline significant. '

12a-i) Imputation techniques to deal with attrition / missing values

Page 8: 'Participants' characteristics at baseline were compared by independent sample t-tests for quantitative variables and by chi-square tests for qualitative variables to detect baseline differences between the control and the intervention group, and to do a drop-out analysis. '

12b) CONSORT: Methods for additional analyses, such as subgroup analyses and adjusted analyses

Page 9: 'All repeated measures ANCOVA's were conducted separately for the total sample, as well as for the at risk sample only (i.e. adults not reaching 10,000 steps a day at baseline). '

RESULTS

13a) CONSORT: For each group, the numbers of participants who were randomly assigned, received intended treatment, and were analysed for the primary outcome

Page 10: flowchart!

Page 9: 'Figure 1 shows the flow of participants through the study. Invitation letters were sent to 1817 people, spread over eight white-collar workplaces. This recruitment process resulted in 274 respondents (response rate of 15.1%) who agreed to participate, of which 137 (50.0%) were allocated to the intervention group and 137 (50.0%) to the control group. Of these, 101 (73.7%) intervention participants and 112 (81.8%) control participants completed one-month post baseline measurements, and 91 (66.4%) intervention participants and 107 (78.1%) control participants completed three-months post baseline measurements. Drop-out analyses indicated that participants from the intervention group ($\chi^2 = 4.661$, P = .03, two-tailed) and commercial companies ($\chi^2 = 27.087$, P < .001, two-tailed) were more likely to drop out. No significant differences were found for demographic variables, pedometer-based and self-reported PA between completers and drop-outs.'

13b) CONSORT: For each group, losses and exclusions after randomisation, together with reasons

Page 10: flowchart

13b-i) Attrition diagram

Not relevant, as the intervention website was not longer available after the intervention period.

14a) CONSORT: Dates defining the periods of recruitment and follow-up

Page 5: 'An invitation e-mail with study information was sent to managers of 18 white-collar workplaces in three waves at different times of the year to overcome seasonal effects. The first wave started in May 2012, the second wave in September 2012 and the third wave in December 2012. '

Page 6: '. One and three months later, all participants again received a blinded pedometer, which was worn for one week. '

14a-i) Indicate if critical "secular events" fell into the study period

Not relevant for this study.

14b) CONSORT: Why the trial ended or was stopped (early)

Not applicable.

15) CONSORT: A table showing baseline demographic and clinical characteristics for each group

Page 11: Table 1

15-i) Report demographics associated with digital divide issues

This information was not provided, as participants were recruited from workplaces, were employees have to work with a pc.

16a) CONSORT: For each group, number of participants (denominator) included in each analysis and whether the analysis was by original assigned groups

16-i) Report multiple "denominators" and provide definitions

Page 14-16: Table 2-4

16-ii) Primary analysis should be intent-to-treat

No intent-to-treat analysis was conducted, as this was already done in the pilot study of De Cocker

17a) CONSORT: For each primary and secondary outcome, results for each group, and the estimated effect size and its precision (such as 95% confidence interval)

Page 14-16: Table 2-4

17a-i) Presentation of process outcomes such as metrics of use and intensity of use

No information provided, as this information is provided in the pilot study of De Cocker.

17b) CONSORT: For binary outcomes, presentation of both absolute and relative effect sizes is recommended

Not applicable.

18) CONSORT: Results of any other analyses performed, including subgroup analyses and adjusted analyses, distinguishing pre-specified from exploratory

Page 11: Table 2-4

18-i) Subgroup analysis of comparing only users

Not relevant, as this was already done by De Cocker et al. in a previous study on the step advice.

19) CONSORT: All important harms or unintended effects in each group

Not applicable, as no harms or unintended effects were present.

19-i) Include privacy breaches, technical problems

Not applicable, as no privace breaches or technical problems occured.

19-ii) Include qualitative feedback from participants or observations from staff/researchers

This information was also available in paper on the pilot study of De Cocker et al.

DISCUSSION

20) CONSORT: Trial limitations, addressing sources of potential bias, imprecision, multiplicity of analyses

20-i) Typical limitations in ehealth trials

Page 18: 'This could possibly be explained by the fact that employers and employees experienced more social support than people invited by their GP, since all employers and employees within a company were invited to participate. Nonetheless, although the response rate was higher than in the study by De Cocker et al. [18], it should be noted that still relatively few people enrolled for the intervention, in comparison with previous computer-tailored intervention studies [12]. Moreover, the recruitment through white-collar workplaces resulted in a selection bias with more highly educated people being involved in the study, which is in line with the outcomes of previous reviews that indicated that mainly higher educated people participate in online interventions [12, 41]. This hampers the generalizability of the study results for those who are less well educated. '
Page 18: 'An unexpectedly high attrition rate was observed in the intervention group as well as in the control group. Almost half of the intervention group participants (43%) and over one third (34%) of the control group participants dropped out at T2, which is relatively high in comparison with the attrition rates reported in recent reviews.'

21) CONSORT: Generalisability (external validity, applicability) of the trial findings

21-i) Generalizability to other populations

Page 18: 'Nonetheless, although the response rate was higher than in the study by De Cocker et al. [18], it should be noted that still relatively few people enrolled for the intervention, in comparison with previous computer-tailored intervention studies [12]. Moreover, the recruitment through white-collar workplaces resulted in a selection bias with more highly educated people being involved in the study, which is in line with the outcomes of previous reviews that indicated that mainly higher educated people participate in online interventions [12, 41]. This hampers the generalizability of the study results for those who are less well educated. '

21-ii) Discuss if there were elements in the RCT that would be different in a routine application setting

Not applicable, nothing would be different in a routine application setting.

22) CONSORT: Interpretation consistent with results, balancing benefits and harms, and considering other relevant evidence

22-i) Restate study questions and summarize the answers suggested by the data, starting with primary outcomes and process outcomes (use)

Page 17: 'The aim of the present study was to evaluate the effectiveness of an online computer-tailored, pedometer-based PA intervention in adults. The results revealed that the step advice has the potential to enhance daily step counts in both the total sample and the at-risk sample. Although, the intervention effects were noticeable in both samples, differences were much more pronounced in the at-risk sample. Moreover, in the at-risk sample a significant intervention effect was also found for self-reported time spent walking. '

22-ii) Highlight unanswered new questions, suggest future research

Page 19: 'more efforts should be devoted to recruit and retain participants in order to improve the public health impact of the intervention. '

Other information

23) CONSORT: Registration number and name of trial registry

The trial is registered by clinicaltrials.gov under the follwing number: NCT02080585

24) CONSORT: Where the full trial protocol can be accessed, if available

The full trial protocol is explained in the paper, however, more information on the intervention website can be found in: De Cocker K, Spittaels H, Cardon G, De Bourdeaudhuij I, Vandelanotte C: Web-based, computer-tailored, pedometer-based physical activity advice: development, dissemination through general practice, acceptability, and preliminary efficacy in a randomized controlled trial. J Med Internet Res 2012, 14:e53.

25) CONSORT: Sources of funding and other support (such as supply of drugs), role of funders

Yes, De Cocker K. was supported by the research foundation Flanders (FWO)

X26-i) Comment on ethics committee approval

Page 6: 'This study protocol was approved by the Ghent University Ethics Committee, and an informed consent was obtained from each participant before the study started. '

x26-ii) Outline informed consent procedures

Page 5: 'Interested employees could sign up by returning a confirmation e-mail to the researchers. On receiving this information, a meeting was organized in each of the eight worksites to deliver all documents for baseline measurement (T0) to the participants, including an informed consent form, a blinded pedometer, an activity log and a self-administered questionnaire. '

X26-iii) Safety and security procedures

Page 6: 'This study protocol was approved by the Ghent University Ethics Committee, and an informed consent was obtained from each participant before the study started. '

X27-i) State the relation of the study team towards the system being evaluated

See page 19: The authors are the developers of the intervention. No other conflicts have been declared.
