# Supplementary material for: Effectiveness of a Web-Based, Computer-Tailored, Pedometer-Based Physical Activity Intervention for Adults: A Cluster Randomized Controlled Trial
Source: J Med Internet Res. 2015 Feb 9;17(2):e38. doi: 10.2196/jmir.3402 (PMC4342625; doi:10.2196/jmir.3402)
Supplement: Supplementary file 2 [file jmir_v17i2e38_app2.pdf]

A.

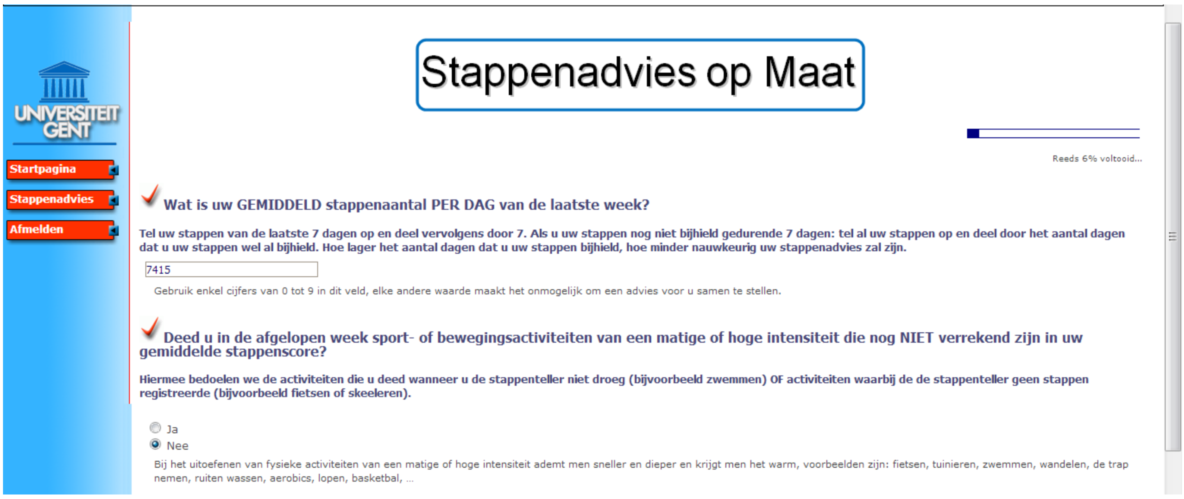

TRANSLATION: TAILORED STEP ADVICE

✓ What is your average daily step count level during the last week?

Please count the number of steps of the last 7 days, and divide this sum by 7. If you did not register steps every day during the last week, please count the number of steps, and divide the sum by the number of days that you did wear the pedometer.

✓ Did you participate in any moderate or vigorous intensity physical activities during the last week, that were not included/registered in your average daily step counts?

These are activities in which you did not wear the pedometer (e.g. swimming), or activities that were not registered by the pedometer (e.g. cycling).

B.

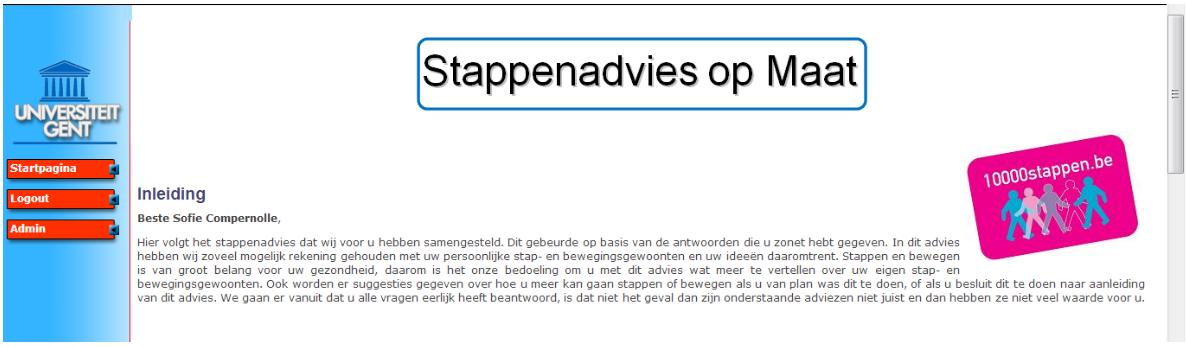

TRANSLATION: TAILORED STEP ADVICE

Introduction

Dear Sofie Compernolle,

Below you will find the step advice that we have developed for you. This step advice was developed based on your answers on the online questionnaire. In the advice, we have taken into account your average daily step counts and other activities, as well as your ideas about this. Being physically active is very important to maintain good health, and that is the reason why we developed this advice for you. We will give you some suggestions to improve your daily step counts. We assume that you answered the questionnaire honestly, if not, this advice will not be accurate and will not be of added value for you.

C.

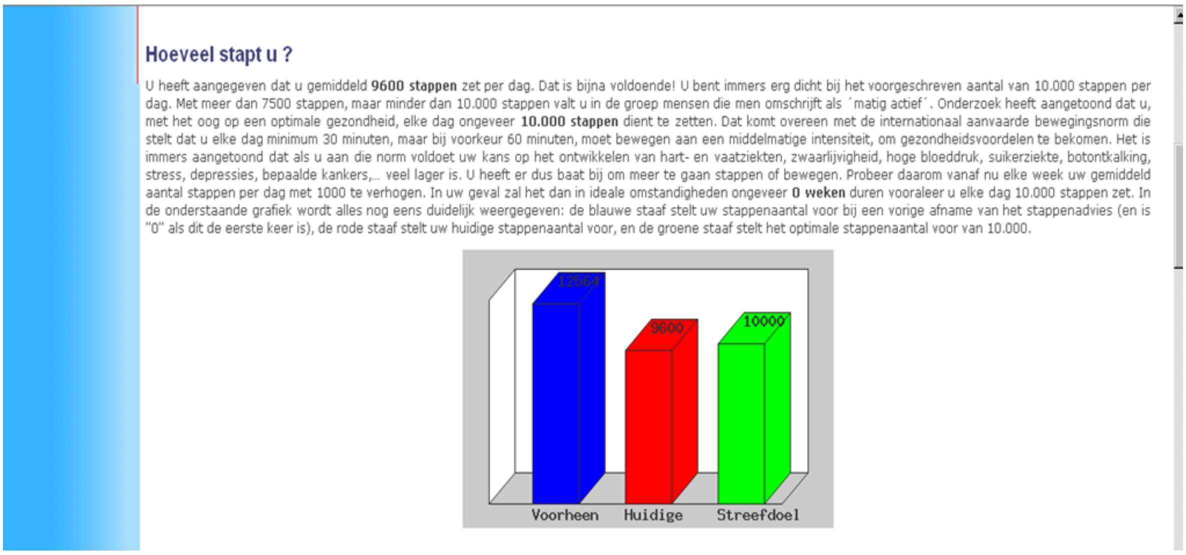

TRANSLATION:

What is your average daily step counts?

You indicated that your average daily step count level is 9,600 steps a day. This is almost enough! You almost reached the recommended guideline of 10,000 steps a day. People who have an average daily step count level between 7,500 and 10,000, are described as being moderately physically active. Research has indicated that you need 10,000 steps a day in order to experience health benefits. This guideline corresponds to the international guideline which recommends at least 30 minutes of moderate intensity physical activity every day. Research has indicated, that if you reach this guideline, you will be less likely to develop cardiovascular diseases, obesity, hypertension, diabetes, osteoporosis, depression, cancer, etc. It is thus beneficial to enhance your daily step counts. Therefore, try to increase your daily step counts with 1,000 steps every week. In your case you will then reach the recommended guideline next week. The graph below shows your average daily step count level (red), your previous average daily step count level (blue) and your goal (green).

D.

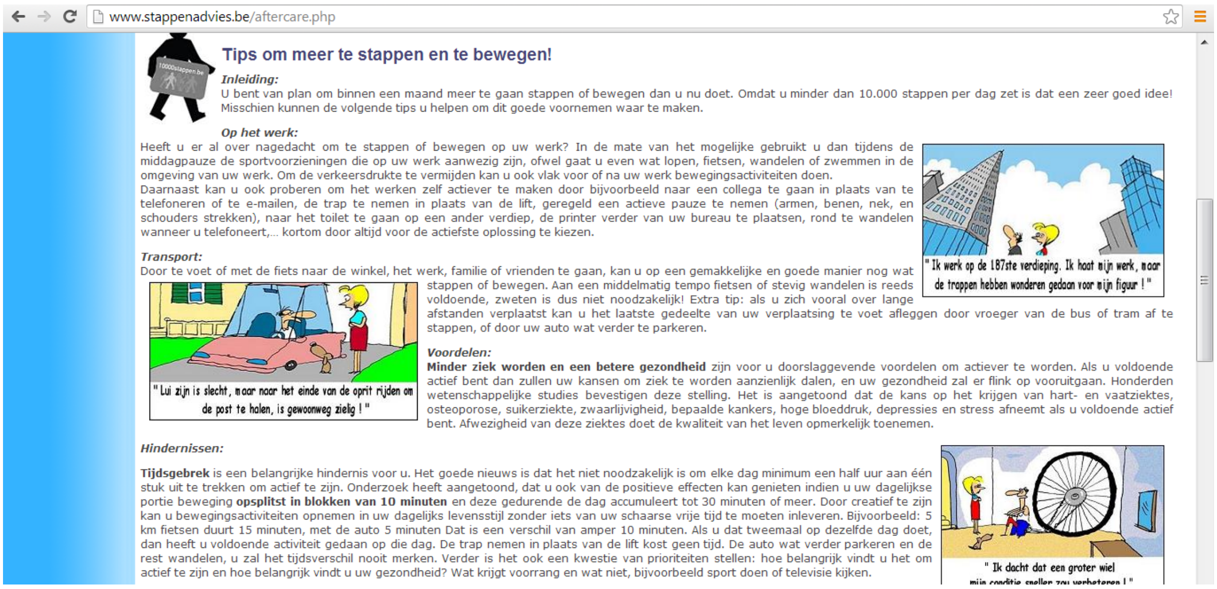

Tips om meer te stappen en te bewegen!

Inleiding:

U bent van plan om binnen een maand meer te gaan stappen of bewegen dan u nu doet. Omdat u minder dan 10.000 stappen per dag zet is dat een zeer goed idee! Misschien kunnen de volgende tips u helpen om dit goede voornemen waar te maken.

Op het werk:

Heeft u er al over nagedacht om te stappen of bewegen op uw werk? In de mate van het mogelijke gebruikt u dan tijdens de middagpauze de sportvoorzieningen die op uw werk aanwezig zijn, ofwel gaat u even wat lopen, fietsen, wandelen of zwemmen in de omgeving van uw werk. Om de verkeersdrukte te vermijden kan u ook vlak voor of na uw werk bewegingsactiviteiten doen.
Daarnaast kan u ook proberen om het werken zelf actiever te maken door bijvoorbeeld naar een collega te gaan in plaats van te telefoneren of te e-mailen, de trap te nemen in plaats van de lift, geregeld een actieve pauze te nemen (armen, benen, nek, en schouders strekken), naar het toilet te gaan op een ander verdiep, de printer verder van uw bureau te plaatsen, rond te wandelen wanneer u telefoneert,… kortom door altijd voor de actiefste oplossing te kiezen.

Transport:

Door te voet of met de fiets naar de winkel, het werk, familie of vrienden te gaan, kan u op een gemakkelijke en goede manier nog wat stappen of bewegen. Aan een middelmatig tempo fietsen of stevig wandelen is reeds voldoende, zweten is dus niet noodzakelijk! Extra tip: als u zich vooral over lange afstanden verplaatst kan u het laatste gedeelte van uw verplaatsing te voet afleggen door vroeger van de bus of tram af te stappen, of door uw auto wat verder te parkeren.

Voordelen:

Minder ziek worden en een betere gezondheid zijn voor u doorslaggevende voordelen om actiever te worden. Als u voldoende actief bent dan zullen uw kansen om ziek te worden aanzienlijk dalen, en uw gezondheid zal er flink op vooruitgaan. Honderden wetenschappelijke studies bevestigen deze stelling. Het is aangetoond dat de kans op het krijgen van hart- en vaatziektes, osteoporose, suikerziekte, zwaarlijvigheid, bepaalde kankers, hoge bloeddruk, depressies en stress afneemt als u voldoende actief bent. Afwezigheid van deze ziektes doet de kwaliteit van het leven opmerkelijk toenemen.

Hindernissen:

Tijdsgebrek is een belangrijke hindernis voor u. Het goede nieuws is dat het niet noodzakelijk is om elke dag minimum een half uur aan één stuk uit te trekken om actief te zijn. Onderzoek heeft aangetoond, dat u ook van de positieve effecten kan genieten indien u uw dagelijkse portie beweging **opsplitst in blokken van 10 minuten** en deze gedurende de dag accumuleert tot 30 minuten of meer. Door creatief te zijn kan u bewegingsactiviteiten opnemen in uw dagelijks levensstijl zonder iets van uw schaarse vrije tijd te moeten inleveren. Bijvoorbeeld: 5 km fietsen duurt 15 minuten, met de auto 5 minuten Dat is een verschil van amper 10 minuten. Als u dat tweemaal op dezelfde dag doet, dan heeft u voldoende activiteit gedaan op die dag. De trap nemen in plaats van de lift kost geen tijd. De auto wat verder parkeren en de rest wandelen, u zal het tijdsverschil nooit merken. Verder is het ook een kwestie van prioriteiten stellen: hoe belangrijk vindt u het om actief te zijn en hoe belangrijk vindt u uw gezondheid? Wat krijgt voorrang en wat niet, bijvoorbeeld sport doen of televisie kijken.

TRANSLATION:

Tips to increase your average daily step counts and physical activity level

Introduction:

You are planning to increase your daily step counts within one month. This is a good idea, as you are currently not meeting the guideline of 10,000 steps a day. Below you will find some tips that can help you to reach the recommended guideline.

At work:

Have you thought about increasing your daily step counts or your physical activity level at work? You can use the sport facilities that are present at work to be physically active during lunch break, or you can go running, cycling or swimming. To avoid traffic, you can be physically active just before or after your working hours. Furthermore, you can try to increase your physical activity level during working hours, e.g. by going to the office of a colleague, instead of sending an e-mail to this colleague, by taking the stairs instead of the elevator, by taking an active break, by using the bathroom on another floor, by placing the printer further away from your desk,… In short, by choosing for the active solution!

Transport:

You can easily increase your physical activity level or your average daily step counts, by choosing active transport to go to your family, to go shopping, to visit friends, to go working etc. Walking or cycling at moderate intensity is already sufficient to experience health benefits, sweating is not necessary. Extra suggestion: if you are covering many kilometers to go to work, you can get off the bus or tram one stop earlier, and walk the last meters/kilometers.

Benefits:

Falling less often ill, and having a better health are your most important benefits to become more physically active. If you are sufficient physically active, than you will be less likely to fall ill. Many scientific studies confirm this statement. It has been shown that the risk for cardiovascular diseases, cancer, osteoporosis, diabetes, obesity, hypertension, depression and stress will decrease if you are sufficiently physically active. This improves the quality of life.

Barriers:

Lack of time is your most important barrier. However, you do not need a block of 30 minutes to be physically active. Research has demonstrated that the beneficial effect of being physically active are also present if you are splitting up your daily physical activities in blocks of 10 minutes. By being creative, you can be physically active for 30 minutes a day, without needing extra time. For example: 5 km cycling takes 15 minutes, with the car it will take 15 minutes. So, the difference is only 10 minutes. If you are doing this twice a day, you reach the international guideline of being physically active for 30 minutes a day. Go up and down stairs, instead of going up with the elevator, will not cost extra time. Furthermore, it is also a matter of priorities; How important is your health? How important is it for you to be physically active?
