# Supplementary material for: Effectiveness of a Web-Based, Computer-Tailored, Pedometer-Based Physical Activity Intervention for Adults: A Cluster Randomized Controlled Trial
Source: J Med Internet Res. 2015 Feb 9;17(2):e38. doi: 10.2196/jmir.3402 (PMC4342625; doi:10.2196/jmir.3402)
Supplement: Supplementary file 3 [file jmir_v17i2e38_app3.pdf]

Multimedia Appendix 3: Comparison of completers and noncompleters

Characteristic	Completers (n=198)	Noncompleters (n=76)	Group comparison	*P*-value
**Demographic variable**				
Gender, n (%)			χ^2=0.20	.63
Male	77 (39.7)	18 (30.5)		
Female	117 (60.3)	41 (69.5)		
Age, mean (SD)	42.3 (11.1)	41.0 (10.8)	t=0.80	.42
BMI, mean (SD)	25.0 (4.0)	25.6 (5.2)	t=0.87	.39
Group, n (%)			χ^2=4.66	.03 [a]
Intervention	91 (46.0)	46 (60.5)		
Control	107 (54.0)	30 (39.5)		
Type of company			χ^2=27.09	<0.01 [a]
For profit	37 (18.7)	38 (50.0)		
Non profit	161 (91.3)	38 (50.0)		
Education, n (%)			χ^2=2.37	.42
Primary/secondary	55 (28.8)	13 (22.4)		
College	93 (48.7)	22 (37.9)		
University	43 (22.5)	23 (39.7)		
Self-rated health, n (%)			χ^2=1.38	.50
Very good/good	155 (80.3)	42 (73.7)		
Fair	31 (16.1)	13 (22.8)		
Very bad/bad	7 (3.6)	2 (3.5)		
Place of residence			χ^2=2.41	.30
Town	39 (20.2)	16 (27.6)		
Outskirts of town	78 (40.4)	25 (43.1)		
Village/countryside	76 (39.4)	17 (29.3)		
**Pedometer-based PA (steps/day), mean (SD)**	8442.6 (3819)	7949.4 (4119)	t=0.86	.39
**Self-reported PA and sedentary time (minutes/day)**				
Sitting time	495.4 (175.3)	502.8 (184.7)	t=0.27	.79
Walking	23.2 (62.2)	24.2 (44.7)	t=0.10	.92
Moderate PA	24.2 (33.3)	25.2 (33.8)	t=0.19	.85
Vigorous PA	9.7 (19.9)	8.6 (18.8)	t=0.37	.71
Total PA	83.0 (122.9)	78.9 (97.1)	t=0.22	.83

[a] $p<0.05$
